# Dietary Phosphorus Requirement for Tambaqui, *Colossoma macropomum*, in the Grow‐Out Phase

**DOI:** 10.1111/jpn.70005

**Published:** 2025-07-17

**Authors:** Ludmila L. C. Menezes, Vânia M. Machado, Cristielle N. Souto, Danilo C. Proença, Guilherme W. Bueno, Igo G. Guimarães

**Affiliations:** ^1^ Laboratório de Pesquisa em Aquicultura Universidade Federal de Jataí, Campus cidade universitária Jataí GO Brazil; ^2^ Departamento de Reprodução Animal e Radiologia Veterinária da FMVZ‐Universidade Estadual de Paulista Botucatu SP Brazil; ^3^ São Paulo State University ‐ UNESP, Aquaculture Center ‐ CAUNESP, Faculty of Agricultural Sciences of Vale do Ribeira Registro SP Brazil

**Keywords:** black cachama, dietary phosphorus, mineral nutrition, mineralization of scales

## Abstract

Phosphorus is essential for fish growth as it is crucial in skeletal development and metabolic reactions. The dietary requirement for this mineral varies among fish species and growth stages. Therefore, the objective of this study was to determine the available phosphorus (AP) requirement for tambaqui during the grow‐out phase (± 400 to 1000 g) using growth parameters, whole‐body macronutrient composition, whole‐body, scales, and bone mineral content, biochemical blood parameters, and activity of antioxidant enzymes as response parameters. A total of 128 tambaqui (395 g ± 20) were distributed across 15 tanks (1000 L each) connected to a recirculating water system, following a completely randomized design with five dietary treatments (4.1, 5.8, 8.0, 9.1, and 10.3 g/kg of AP) and three replicates per treatment. The fish were fed with experimental diets to apparent satiation for 180 days. The apparent digestibility coefficients (ADC) of the experimental diets were also determined to report the requirement on an available nutrient basis. No mortality or apparent signs of P deficiency were observed during the growth trial. The ADC of the diets decreased with increasing total phosphorus levels. AP supplementation in the diet did not affect growth performance parameters except phosphorus utilization, which decreased linearly with increasing AP supplementation. Bone mineralization increased with dietary AP supplementation, while the scale and whole‐body mineralization were unaffected. Dietary AP levels, except for serum phosphorus and triglycerides, significantly affected blood biochemical parameters. The highest superoxide dismutase (SOD) activity was observed in fish fed 8 g/kg AP in the diet. The estimated requirement for the highest serum immunoglobulin concentration was 6.17 g/kg of AP. Our findings suggest that tambaqui in the grow‐out stage can develop adequately without inorganic phosphorus supplementation to plant‐based diets; 4.1 g/kg AP (or 25.2 mg AP/kg BW^0.8^/day) seems to be sufficient to maintain growth parameters. However, this minimal level is associated with increased adiposity. To maximize bone mineralization, 10.3 g/kg AP (or 63.5 mg AP/kg BW^0.8^/day) is required. Intermediate dietary levels (around 6.17 g/kg AP or 37.9 mg AP/kg BW^0.8^/day) were associated with higher total serum protein, lysozyme, and immunoglobulin concentrations, suggesting possible physiological benefits.

## 1 Introduction

Phosphorus (P) is one of the most essential macrominerals for fish nutrition (Sugiura and Ferraris 2004). This mineral plays a fundamental role in bone development, bioenergetics, and intermediary metabolism, affecting the use of nutrients in energy metabolism (Sugiura and Ferraris 2004; Xie et al. 2017; Lu et al. 2017). Several studies have demonstrated that P deficiency leads to suppressed growth, low bone mineralization, and compromised immune functions (Fjelldal et al. 2016; Chen et al. 2018). Although fish can absorb phosphorus from water, its concentration in freshwater is relatively low to meet the nutritional requirements of fish raised in intensive or semi‐intensive systems, and diets are the primary source of phosphorus for these fish (NRC 2011).

On the other hand, the excess phosphorus excreted by cultured fish can stimulate the eutrophication of water bodies and cause significant environmental impacts throughout the aquatic food chain. Despite not being the scarcest nutrient in freshwater ecosystems, phosphorus is the most limiting factor for primary production. It is considered a key indicator in studies of carrying capacity and water quality (Green et al. 2002), as its occurrence in the aquatic environment is lower than or equal to its demand for the growth of photosynthetic organisms. As a result, the releases of phosphorus into freshwater are alarming and can be predicted (Bueno et al. 2023), while the removal of soluble phosphorus in water bodies is complex (Moody et al. 2015). However, commercial fish feeds often consider only the total phosphorus fraction. In contrast, the content of available phosphorus (AP) varies depending on the quality of ingredients and the sources of phosphorus used in aquaculture diets (Morales and Moyano 2010).

Reducing the phosphorus content in fish diets alone would not be a viable solution to decrease phosphorus excretion in the aquatic environment. The critical factor in minimizing phosphorus discharge generated by aquaculture is adjusting the dietary intake of available phosphorus (AP) to meet the strict demands of the fish according to the growth stage. Phosphorus requirements are altered due to physiological and environmental factors and tend to decrease as the animal grows (Lellis et al. 2004). However, data on nutritional requirements are scarce, as studies on mineral nutrition for tambaqui remain limited (Guimarães and Martins 2015). So far, only three studies have reported the phosphorus requirement for tambaqui in the initial phase (± 17 g) (Menezes et al. 2021), fingerlings (± 0.51 g) (Sousa et al. 2019), and juveniles (± 144 g) (Araújo et al. 2017). To our knowledge, no data on the phosphorus requirement for tambaqui in the grow‐out phase are available.

Given the information mentioned above and the lack of data on dietary phosphorus requirements for tambaqui in different growth phases, this study aimed to determine the requirement for available phosphorus in the grow‐out phase (400–1000 g) of tambaqui. Therefore, this study used growth performance parameters, body composition, body, bones and scales mineralization, hematological and biochemical blood profiles, and antioxidant enzyme activity as response variables to determine the P requirement.

## 2 Materials and Methods

All the experimental procedures were approved by the Ethics Committee on Animal Use of the Universidade Federal de Jataí (process number 014/2020), which follows the guidelines for handling animals proposed by the National Council for the Control of Animal Experiments (CONCEA).

### 2.1 Preparation of the Experimental Diets

Five diets were formulated to meet the minimum requirements for tambaqui with 13.10 MJ/kg digestible energy and 300 g/kg digestible protein (Guimarães and Martins 2015; Oliveira 2019). Potassium phosphate was used as the primary inorganic P source to reach the following dietary AP levels: 4.1, 5.8, 8.0, 9.1, and 10.3 g/kg diet (Araújo et al. 2017). In addition, all diets were supplemented with 5 g/kg of chromium oxide III as an inert digestibility marker for nutrient digestibility evaluation. Limestone was used to adjust the calcium levels and maintain the same Ca:P ratio in all experimental diets (Table 1). Ca and P sources were used at the expense of the inert ingredient kaolin to keep the nutrient content of the diets more similar and maintain a similar Ca:P ratio.

**Table 1:** Experimental diets and chemical composition of the experimental feeds.

Ingredients (g/kg)	Diets				
	4.1	5.8	8	9.1	10.3
Soybean meal	287	287	287	287	287
Corn meal	165	165	165	165	165
Soy protein concentrate	152	152	152	152	152
Rice bran	90	90	90	90	90
Wheat bran	96	96	96	96	96
Corn gluten meal	76	76	76	76	76
Cellulose	18	18	18	18	18
Vitamin C	5.0	5.0	5.0	5.0	5.0
NaCl	1.0	1.0	1.0	1.0	1.0
l‐Lysine	3.9	3.9	3.9	3.9	3.9
DL‐Methionine	6.5	6.5	6.5	6.5	6.5
Tryptophan	0.6	0.6	0.6	0.6	0.6
Vitamin/mineral premix^a^	2.5	2.5	2.5	2.5	2.5
BHT^b^	0.2	0.2	0.2	0.2	0.2
Soybean oil	16	16	16	16	16
Limestone	2.3	7.0	11.9	16.8	21.7
Kaolin	78.3	63.5	48.1	32.7	17.3
Potassium phosphate	0.00	10.1	20.6	31.1	41.6
**Chemical composition (g/kg of dry matter)**					
Crude protein	323	342	358	342	324
Digestible protein^c^	305	307	296	312	273
Fat	46	51	51	54	50
Ash	122	122	127	133	158
Calcium	7.2	10.3	11.6	16.5	18.1
Total phosphorus	4.9	7.1	9.6	11.5	13.5
Available phosphorus^c^	4.1	5.8	8.0	9.1	10.3
Ca:P ratio	1.47	1.46	1.21	1.44	1.34

^a^ Guaranteed levels of the Supplement (IU or mg/kg diet): vitamin A, 16060; vitamin D3, 4510; vitamin E (DL‐α tocopherol), 250; vitamin K (menadione sodium bisulfite), 30; thiamine, 32; riboflavin, 32; Ca‐d‐pantothenate, 80; niacin, 170; biotin, 10; folic acid, 10; cyanocobalamin, 0.032; pyridoxine, 32; Na_2_SeO_3_, 0.7; MnO, 50; ZnO, 150; FeSO_4_, 150; CuSO_4_, 20; CoSO_4_, 0.5; I_2_Ca, 1.0 (Guabi Nutrição Animal Ltda).

^b^ Butylated hydroxytoluene ‐ antioxidant.

^c^ Values obtained from the analyzed total P values and the digestibility coefficients of P obtained in the digestibility trial (Appendix 1).

The ingredients were weighed and mixed. Then, a premixture with micronutrients was prepared to facilitate incorporation. Oil and water were then added to produce a homogeneous mash. The mash was extruded in a single‐screw laboratory extruder (Exteclab, Extec) to obtain pellets suitable for the fish size. Finally, the diets were oven‐dried at 55°C for 24 h and stored in plastic bags at −20°C until used.

### 2.2 Animals, Facilities, and Experimental Design

The experiment was completely randomized, with five treatments (dietary available phosphorus levels) and three replications comprising 15 experimental units. The experimental unit was a 1000 L tank containing eight fish.

The fish were acquired from a commercial fish farm and underwent a 15‐day adaptation period, consuming the control diet (4.1 g/kg of available phosphorus, AP). A homogeneous batch of 128 tambaqui with an average body weight of 395 ± 20 g was selected, and 120 fish were stocked in 15 1000‐L polyethylene tanks connected to a recirculating water system with physical and biological filtration and temperature control. The remaining fish were used for the initial whole‐body chemical composition. Water quality parameters were maintained within the optimum range for tambaqui (temperature: 28 ± 2°C; pH: 7; ammonia: 0.25 mg/L; dissolved oxygen: 6 ± 1 mg/L). Water exchange maintained the dissolved P concentration below 0.5 mg/L throughout the trial.

Fish were fed to apparent satiation twice daily (08:00 and 16:00), 6 days a week, for 180 days. The feed consumed was recorded daily, while fish were weighed monthly. The following growth parameters were determined: survival rate, final weight (FW), daily feed intake (DFI), daily weight gain (DWG), feed conversion ratio (FCR), phosphorus utilization rate (PUR), and protein efficiency ratio (PER) according to the equations below:

- Daily weight gain (DWG) = (Final weight (g) − Initial weight (g))/number of days
- Daily feed intake (DFI) = Feed quantity (g)/fish biomass (g)/number of days
- Apparent feed conversion ratio (FCR) = Feed intake (g)/weight gain (g)
- Phosphorus utilization rate (PUR) = [(Weight gain (g)/phosphorus intake (g)) × 100]
- Protein efficiency ratio (PER) = Weight gain (g)/protein intake (g)

A different trial was performed only to determine the ADC of nutrients of the experimental diets and report P content of the experimental diets on a digestible basis. Data and description of the procedure are shown in Appendix 1.

For all experimental procedures, the fish were pre‐anesthetized with an alcoholic solution of Eugenol (0.01 ml/L) (Roubach et al. 2005). The animals designated for biological sample collection were euthanized by a megadose of the anesthetic (1 ml/L).

### 2.3 Chemical Composition

Before the start of the experimental period, six fish were randomly sampled for whole‐body composition analysis. At the end of the 180 days, all fish were weighed and sampled for tissue analysis after a 12‐h fasting period. In addition, two fish from each tank were collected for whole‐body chemical composition.

The diets and fish samples were analyzed in duplicate for chemical composition. Moisture content was determined by drying in a 105°C oven for 24 h. Crude protein (N * 6.25) was analyzed using the Kjeldahl method; total lipids were determined using a Soxhlet fat extractor, and ash content was determined by incineration in a muffle furnace at 550°C for 5 h (AOAC 2000).

Eight fish from each treatment were dissected to collect operculum bones, head bones, vertebrae, and scales for mineral composition. The bones were washed with deionized water to remove any remaining muscle tissue and then defatted in a chloroform and methanol solution (1:1). The material was dried in an oven for 24 h following the procedure described by Roy et al. (2002). The samples were then digested with nitric and perchloric acids to determine mineral content in the diets, feces, whole body, bones, and scales. After digestion, the extracts were diluted in 50 ml of deionized water, and the phosphorus concentrations were determined in a spectrophotometer at 420 nm. Calcium (Ca), magnesium (Mg), zinc (Zn), and manganese (Mn) concentrations were determined by atomic absorption spectrometry. These analyses were performed following the procedures outlined in AOAC (2000).

### 2.4 Blood Biochemistry and Enzymes

Blood samples were collected from four fish in each tank at the end of the experimental period. One milliliter syringe without anticoagulant was used for collecting blood for serum analysis. The blood samples were collected by puncturing the caudal vessel. The serum samples were centrifuged at 4000 rpm for 10 min at four °C and then stored in liquid nitrogen.

The serum samples were thawed in a refrigerator and homogenized to analyze blood chemistry and enzyme activity. Total serum phosphorus and calcium levels, triglycerides, proteins, albumin, and serum alkaline phosphatase were determined using a photometric kit (*Labtest*). All the described variables were analyzed using an automated biochemical analyzer (Wiener lab group, Rosario, Argentina).

The lysozyme activity was determined according to Demers and Bayne (1997) and modified for use with tambaqui serum. The analysis is based on the lysis of *Micrococcus lysodeikticus* suspension, using lysozyme from chicken egg whites as a standard. The concentration of total immunoglobulin (Ig) was determined according to Siwicki et al. (1993) using the total protein kit (Labtest) following the Biuret methodology. The superoxide dismutase (SOD) activity was quantified using a microplate reader at 570 nm (De Barrios Freitas et al. 2017).

### 2.5 Statistical Analysis

All data were checked for normality (Shapiro–Wilk test) and variance homogeneity (Bartlett's test). Then, linear and Nonlinear (quadratic, linear response plateau, and curvilinear response plateau) regression models were tested to estimate the requirement for available phosphorus. The choice of the model was based on the R^2^, P‐value, and mean square error of residuals. Additionally, a lack‐of‐fit test was performed for all regression analyses. For variables that showed a significant quadratic regression model, 95% of the maximum response was calculated and reported as the requirement for the specific response parameter (Bertocchi et al. 2019). When no regression models could be fitted, data were subjected to analysis of variance and compared using the Student Newman Keuls (SNK) or Duncan multiple range tests for blood biochemistry parameters. A significance level of *p* ≤ 0.05 was used for all statistical analyses. The data were analyzed using the R software, and graphs were prepared using GraphPad Prism.

## 3 Results

Except for phosphorus utilization, which linearly decreased with increasing dietary AP levels, all growth performance parameters were not affected (*p* > 0.05) (Table 2). No mortality was observed during the experimental period.

**Table 2:** Final weight (FW), daily feed intake (DFI), daily weight gain (DWG), feed conversion ratio (FCR), phosphorus utilization rate (PUR) and protein efficiency rate (PER) of tambaqui fed diets containing graded available P (AP) levels for 180 days (*n* = 3).

Variables	Dietary AP levels (g/kg)					*p* values		
	4.1	5.8	8	9.1	10.3	LR^b^	NLR^c^	ANOVA
FW (g)	912 ± 49	986 ± 67	932 ± 11	936 ± 56	927 ± 48	0.827	0.327	0.989
DFI (g)	5.7 ± 0.4	6.2 ± 1.2	6.7 ± 0.6	5.8 ± 0.7	5.8 ± 1.1	0.926	0.462	0.610
DWG (g)	2.9 ± 0.3	3.3 ± 0.4	3.1 ± 0.2	3.0 ± 0.3	3.0 ± 0.9	0.942	0.586	0.731
FCR	2.0 ± 0.1	1.9 ± 0.2	2.1 ± 0.1	1.9 ± 0.1	2.0 ± 0.3	0.935	0.253	0.822
PUR^a^ (%)	1.8 ± 0.1	1.3 ± 0.2	0.9 ± 0.1	0.8 ± 0.1	0.7 ± 0.1	< 0.001	0.016	< 0.001
PER (%)	2.2 ± 0.2	2.2 ± 0.3	2.0 ± 0.1	2.0 ± 0.2	2.2 ± 0.2	0.613	0.432	0.542

*Note:* Each data is a mean ± standard deviation of 3 aquaria containing 12 fish. Means in a row followed by the same superscript letter are not statistically different by SNK multiple range test (*p* > 0.05).

^a^ Linear = 2.421087‐0.177693x (R^2^ = 0.92).

^b^ LR = Linear Regression.

^c^ NLR = Nonlinear Regression.

The increase in available phosphorus levels in the diet only affected the phosphorus digestibility coefficient, which decreased linearly with increasing phosphorus levels (Appendix 1).

The carcass lipid content decreased until it reached a plateau at 5.59 g/kg AP. In contrast, the moisture content showed a quadratic response, increasing until reaching a maximum level at 7.46 g/kg AP and decreasing afterward. However, AP supplementation did not affect the ash and crude protein contents in the whole body (Table 3).

**Table 3:** Whole‐body chemical composition (dry matter basis) of tambaqui fed diets containing graded available P (AP) levels for 180 days (*n* = 6).

Variables (g/kg)	Dietary AP levels (g/kg)					*p* values		
	4.1	5.8	8	9.1	10.3	LR^c^	NLR^d^	ANOVA
Moisture^a^	375 ± 14	498 ± 15	527 ± 47	427 ± 21	447 ± 23	0.110	< 0.001	< 0.001
Ash	114 ± 8	115 ± 10	102 ± 27	105 ± 10	108 ± 15	0.265	0.596	0.531
Protein	160 ± 38	201 ± 3	200 ± 17	207 ± 20	195 ± 18	0.055	0.145	0.076
Fat^b^	385 ± 29	208 ± 28	263 ± 32	217 ± 16	205 ± 21	< 0.001	< 0.001	< 0.001

*Note:* Means **± _**SD are derived from two determination per fish, two fish per tank and three tanks per treatment. Means in the rows followed by different letters differ significantly according to the Student–Newman–Keuls (SNK) test. (*P* < 005) (*n* = 6). (*p* > 0.05).

^a^ Quadratic regression: Whole‐body moisture = −103.6 + 164.5x – 11.02x^2^ (R^2^ = 0.58, Preq = 7.08 g/kg).

^b^ Linear Response plateau: Whole‐body fat = 233.3−2.89 (x−5.59) (R^2^ = 0.81, Preq = 5.59 g/kg).

^c^ LR = Linear Regression.

^d^ NLR = Nonlinear Regression.

Although a significant effect for the Nonlinear models was observed for Zn content in the whole body, and Ca, Mg, and Zn in the scales, no biological model fitted to these data (Table 4). The phosphorus concentration increased linearly in the whole body and vertebrae. However, dietary AP levels did not affect P concentration in the scales. The calcium content in the entire body linearly increased until reaching a plateau at 7.84 g/kg AP. On the other hand, the Ca content in the different body compartments was not significantly affected by the dietary AP levels. Except for the Mg content in the scale, Mg and Mn content in the body compartments evaluated in this study were not affected by the dietary AP levels (Table 4). The highest Mg content in the scales was observed in fish‐fed diets containing 9.1 g/kg AP. Despite the body compartments' Zn content being generally unaffected by the dietary AP levels, a higher Zn concentration in the whole body was observed in fish‐fed diets containing 8 g/kg (Table 4).

**Table 4:** Whole‐body, vertebrae, and scales mineral composition of tambaqui fed diets containing graded available P (AP) levels for 180 days (*n* = 6).

Variable	Dietary AP levels (g/kg)					*p* values		
	4.1	5.8	8	9.1	10.3	LR^4^	NLR^5^	ANOVA
**Whole‐body**								
P^1^ (g/kg)	18.1 ± 6.5	26.2 ± 8.3	32.9 ± 10.7	28.1 ± 2.7	36.1 ± 10.3	0.011	0.454	0.064
Ca (g/kg)^2^	35.0 ± 10.7	51.9 ± 3.2	74.9 ± 13.1	43.2 ± 11.1	41.1 ± 10.3	0.832	< 0.001	< 0.001
Ca:P ratio	1.93	1.98	2.28	1.54	1.14			
Mg (g/kg)	3.1 ± 1.9	3.3 ± 0.3	3.9 ± 0.7	2.6 ± 0.7	2.3 ± 0.7	0.173	0.305	0.252
Zn (mg/kg)	58.3^ab^ ± 11.2	54.1^ab^ ± 9.4	81.5^a^ ± 24.4	30.3^b^ ± 15.6	53.2^ab^ ± 25.9	0.265	0.017	0.024
Mn (mg/kg)	188 ± 66	263 ± 30	308 ± 42	254 ± 70	248 ± 75	0.254	0.117	0.133
**Vertebrae**								
P^3^ (g/kg)	180 ± 22	205 ± 21	208 ± 13	214 ± 17	225^a^ ± 24	< 0.001	0.636	0.011
Ca (g/kg)	252 ± 65	226 ± 48	237 ± 74	234 ± 91	227 ± 42	0.698	0.971	0.098
Ca:P ratio	1.41	1.10	1.14	1.09	1.01			
Mg (g/kg)	25.8 ± 8	21.0 ± 2.4	24.2 ± 14.3	19.8 ± 9.6	32.0 ± 8.2	0.460	0.337	0.410
Zn (mg/kg)	543 ± 80	472 ± 54	491 ± 176	435 ± 159	551 ± 142	0.924	0.543	0.695
Mn (g/kg)	2.0 ± 1.3	1.4 ± 0.2	1.5 ± 0.9	1.30 ± 0.2	1.9 ± 1.3	0.841	0.634	0.772
**Scale**								
P (g/kg)	171 ± 23	174 ± 17	183 ± 20	160 ± 21	165 ± 14	0.296	0.310	0.324
Ca (g/kg)	184 ± 39	215 ± 56	156 ± 31	262 ± 89	202 ± 64	0.292	0.044	0.058
C:P ratio	1.08	1.26	0.85	1.63	1.22			
Mg (g/kg)	36^ab^ ± 14	41^ab^ ± 14	21^c^ ± 7	50^a^ ± 18	35^b^ ± 9	0.760	0.042	0.075
Zn (g/kg)	3.1 ± 0.6	4.2 ± 1.5	2.1 ± 1.6	4.6 ± 1.1	2.9 ± 0.7	0.999	0.030	0.056
Mn (mg/kg)	841 ± 327	774 ± 306	438 ± 163	771 ± 183	569 ± 147	0.167	0.159	0.143

*Note:* Means **± _**SD are derived from two determination per fish, two fish per tank and three tanks per treatment. Means in a row followed by the same superscript letter are not statistically different by SNK multiple range test (*p* > 0.05).

^1^ Linear effect equation: Whole‐body P = 9.827 + 2.474x (R^2^ = 0.81).

^2^ Broken line linear effect equation: Whole‐body calcium = −5.02205 + 9.796351(x‐x_0_) (x_0_ = 7.8 g/kg, R^2^ = 0.88).

^3^ Linear effect equation: Vertebrae P = 159.7 + 6.264x (R^2^ = 0.89).

^4^ LR = Linear Regression.

^5^ NLR = Nonlinear Regression.

**Figure 1:**
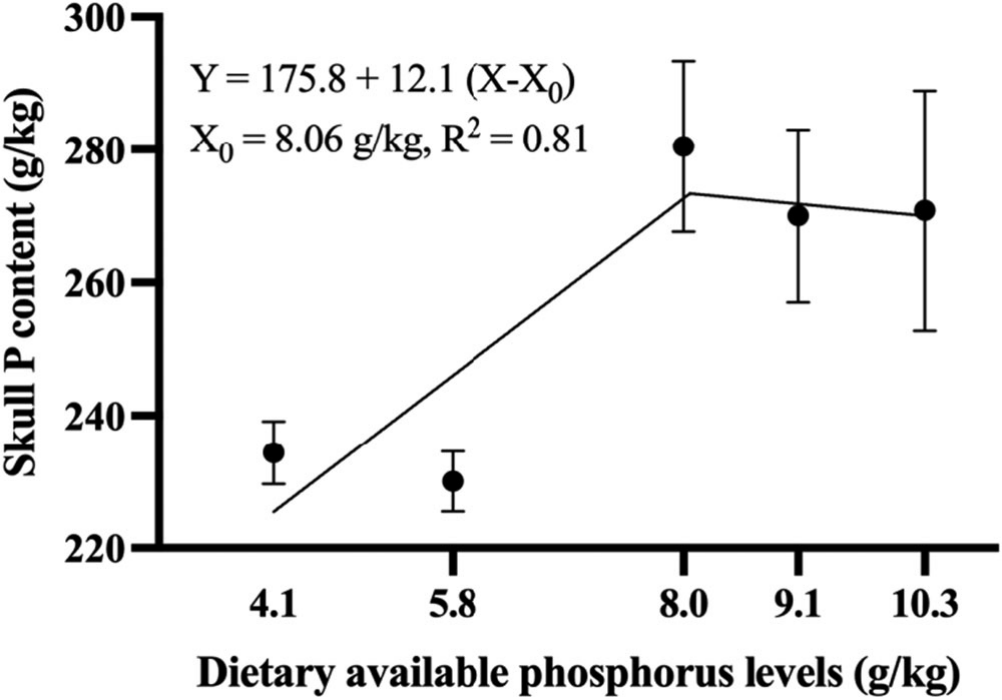
Skull P content of tambaqui fed diets containing graded levels of dietary digestible P. Each point represents the means **± **SD of six replicates (fish).

A segmented linear regression model provided the best fit for the phosphorus concentration in the skull. According to the regression analysis, the estimated requirement for efficient phosphorus deposition in the skull was 8.06 g/kg AP in the diet (Figure 1).

**Figure 2:**
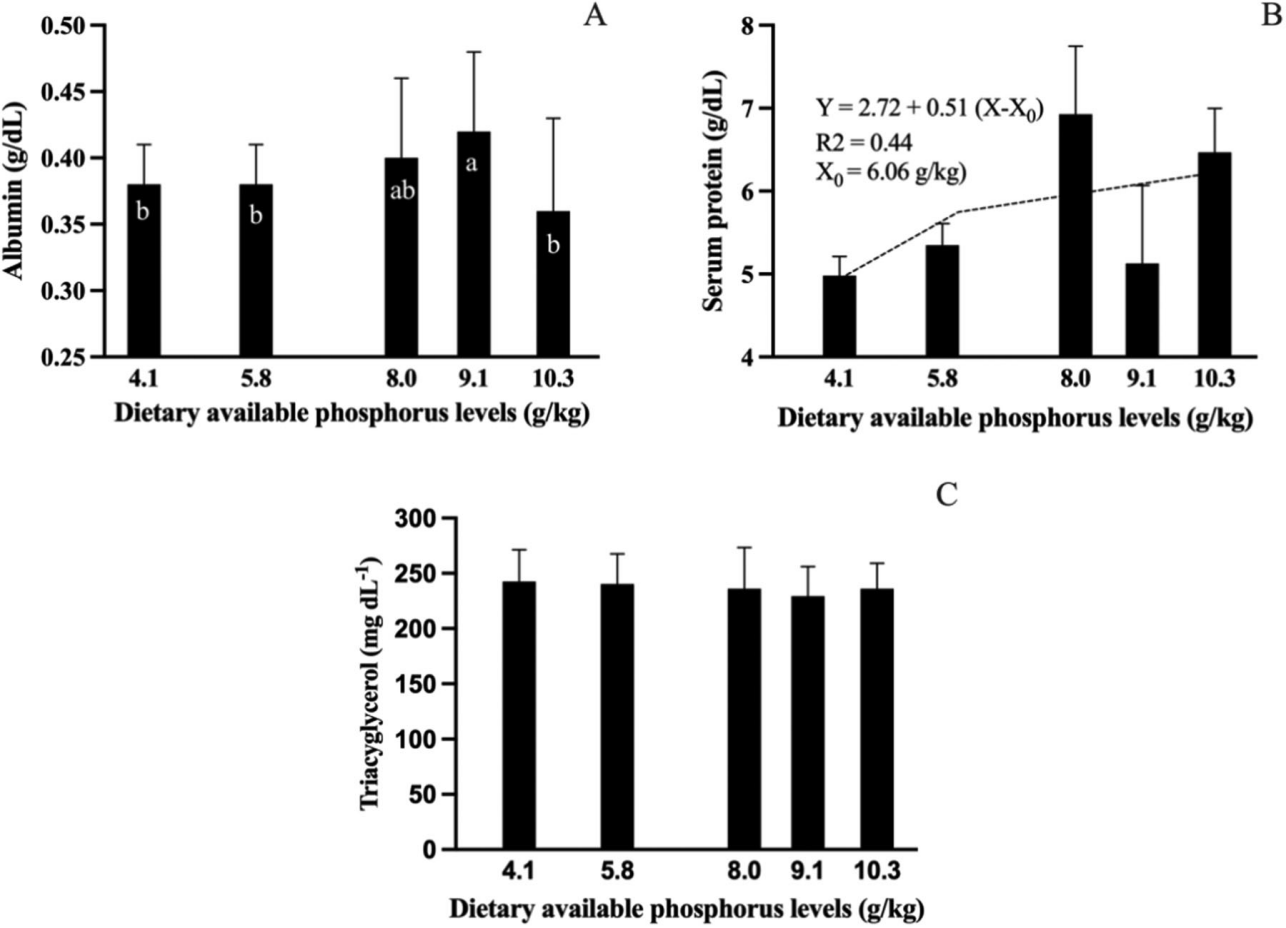
Albumin (A), total proteins (B), triglycerides (C) of tambaquis fed with different levels of available phosphorus (*n* = 12). Values are means **± **SD of two determinations per fish, four fish per tank and three tanks per treatment. Bars with the same lowercase letter do not differ significantly (*p* > 0.05) according to the Duncan's multiple range test. Best fitted model according to Akaik's Information Criteria was the Linear broken line model (*p* = 0.035).

Dietary AP levels significantly affected all the biochemical blood parameters except triglycerides (Figure 2C). Fish fed the diet containing 9.1 g/kg AP showed a higher albumin concentration (Figure 2A). Total serum protein linearly increased in response to dietary AP levels up to 6.06 g/kg (Figure 2B).

**Figure 3:**
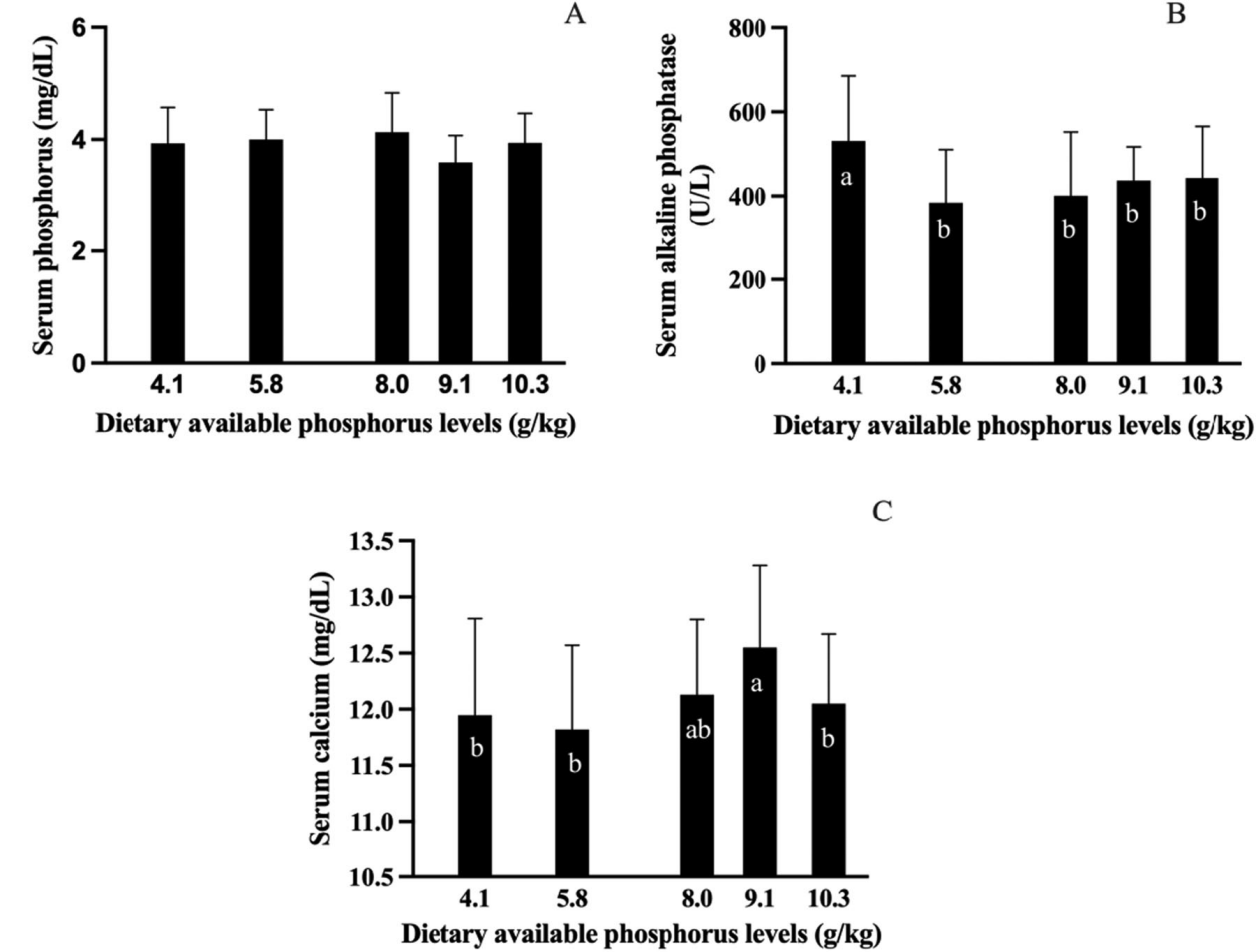
Total serum phosphorus (A), alkaline phosphatase activity (B), and total serum calcium (C) of tambaquis fed with different levels of available phosphorus (*n* = 12). Values are means **± **SD of two determinations per fish, four fish per tank, and three tanks per treatment. Bars with the same lowercase letter do not differ significantly (*p* > 0.05) according to the Duncan's multiple range test.

Dietary phosphorus levels did not affect the serum phosphorus concentration (Figure 3A). However, alkaline phosphatase activity significantly decreased in all AP‐supplemented groups. The highest enzyme activity was observed in fish fed the non‐supplemented diet (4.1 g/kg) (Figure 3B). In addition, fish fed the diet containing 9.1 g/kg AP showed a higher serum calcium concentration (Figure 3C).

**Figure 4:**
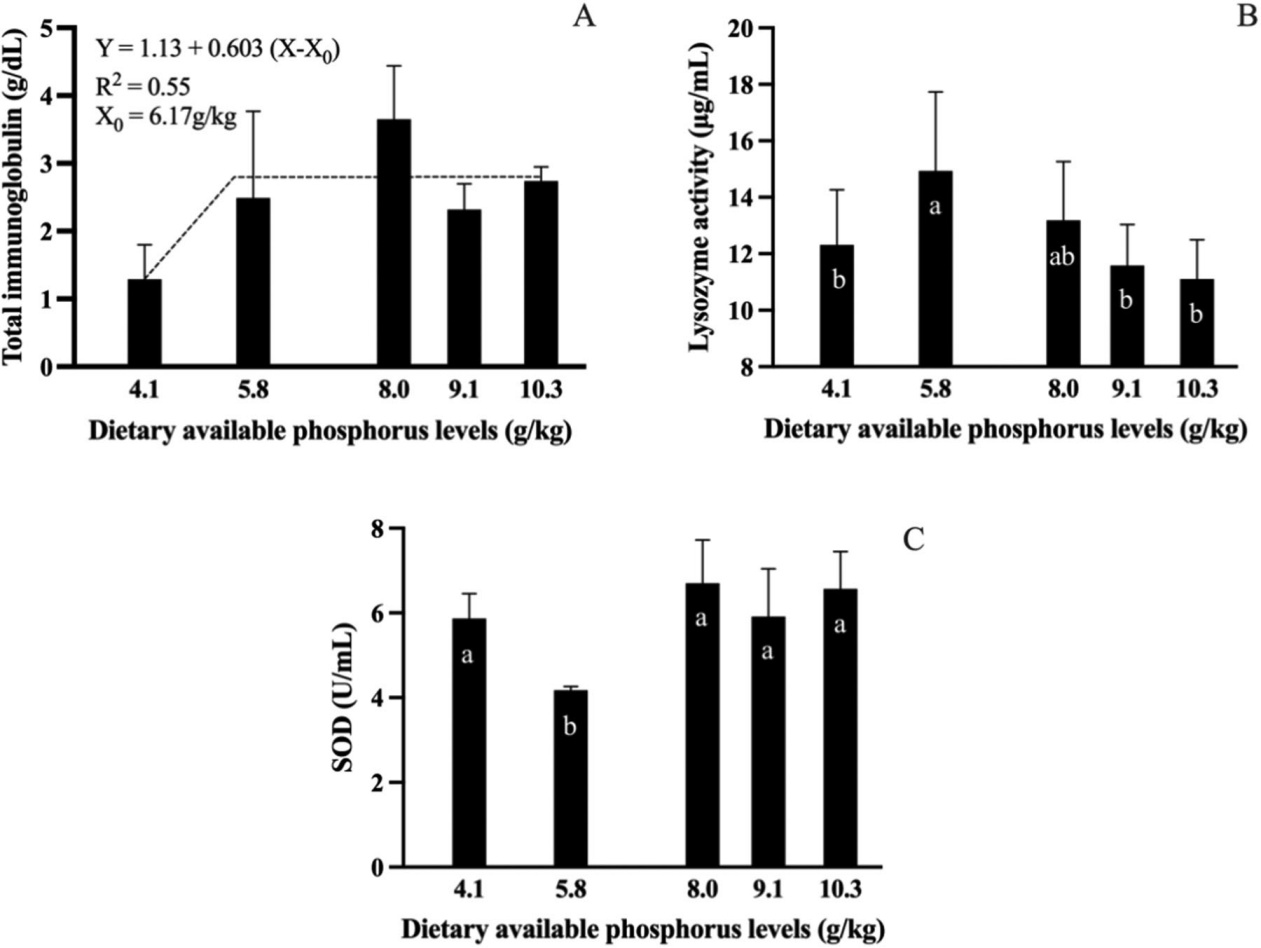
Total immunoglobulin (A), lysozyme activity (B), and SOD (C) of tambaquis fed with different levels of available phosphorus (n = 12). Values are means **± **SD of two determinations per fish, four fish per tank and three tanks per treatment. Bars with the same lowercase letter do not differ significantly (*p* > 0.05) according to the Duncan's multiple range test. Best fitted model for total immunoglobulin according to Akaik's Information Criteria was the Linear broken line model (*p* = 0.019).

The segmented linear regression model best fitted to total immunoglobulin data (Figure 4A). Immunoglobulin increased with dietary AP levels and plateaued at 6.17 g/kg diet. Fish‐fed diets containing 5.8 g/kg AP showed a decrease in SOD activity, while lysozyme activity was higher in this group (Figure 4B and C).

## 4 Discussion

Using a recirculating aquaculture system (RAS) in this study provided a controlled environment to minimize external variables and focus on the dietary effects. However, the RAS conditions, such as enhanced water filtration and reduced environmental variability, could have influenced nutrient utilization, particularly phosphorus metabolism, as the system tends to accumulate soluble phosphorus in the water. Therefore, we monitored the total P content in the water and limited its level to 0.5 mg/L, aiming to reduce the influence of water phosphate levels on P metabolism. These factors should be considered when applying the findings to other production systems with different management practices.

Despite this controlled environment, the available phosphorus (AP) requirement usually decreases according to the developmental stage of the fish (Lellis et al. 2004). In this study, tambaqui in the grow‐out stage (± 950 g) fed P‐unsupplemented plant‐based diets (4.1 g/kg AP) exhibited average growth and development, without any signs of mineral deficiency. Additionally, no effect of P supplementation on growth was observed in this trial. Therefore, 4.1 g/kg AP might be enough to support the growth of tambaqui in this size range. Although phosphorus needs in fish nutrition are usually given as a dietary concentration (g/kg feed), this measure does not consider differences in feed intake or energy density. To better reflect physiological relevance, we estimated phosphorus intake based on metabolic body weight. Considering the average feed intake from each dietary group during the trial and the fish's final body weigh (BW)t range (from 0.912 to 0.986 kg), phosphorus intake across treatments was estimated to be from 25.2 to 63.5 mg of AP per kg^0.80^ BW daily. Accordingly, the P requirement estimate for supporting growth of tambaqui was 25.2 mg AP/kg BW^0.8^/day. These estimates matches intake levels and requirements based on growth reported for other freshwater fish species under similar conditions (NRC 2011; Antony Jesu Prabhu et al. 2013).

Higher AP requirements based on weight gain have been reported for other growth stages of tambaqui. For instance, the AP requirement for fry (± 0.51 g) and juveniles (± 17 to 150 g) was estimated to be 7.1 (Sousa et al. 2019) and 6.6 g/kg (Menezes et al. 2021), respectively. On the other hand, the lack of effect of P supplementation on fish growth has been reported for tambaqui in the juvenile stage (± 144 g) (Araújo et al. 2017), as well as for other aquaculture fish species such as pacu (*Piaractus mesopotamicus*) (Diemer et al. 2011; Signor et al. 2011), largemouth bass (*Micropterus salmoides*) (Miller et al. 2021), taimen (*Hucho taimen*) (Wang et al. 2017), and Senegalese sole (*Solea senegalensis*) (Salas‐Leiton et al. 2017). Although there was no significant difference in feed intake among fish fed the different dietary treatments in this study, larger fish displayed agonistic behavior during feeding. In contrast, smaller ones exhibited submissive responses such as fleeing. This led to a high variation in our feed intake data for some fish groups.

It is also important to note that the phosphorus source used in this study was monopotassium phosphate (KH₂PO₄), which supplies both phosphorus and potassium. Although potassium is typically not seen as a limiting nutrient in commercial aquafeeds, some studies suggest it can influence growth and physiological responses in freshwater fish (Shiau and Hsieh 2001). While the current experimental design did not allow us to isolate the effects of potassium and no growth improvements were observed with increasing AP levels, we recommend that future studies specifically investigate the interaction between dietary available phosphorus levels and phosphorus sources containing potassium.

P digestibility was high in all diets, decreasing with increasing levels of total P. Concurrently, the P utilization rate was more efficient in fish‐fed low AP levels. Similarly, a previous study observed the same response when feeding tambaqui juveniles (± 144 g) with increasing P levels in plant‐based diets (Araújo et al. 2017). The P digestibility for tambaqui is generally higher than that reported for other omnivorous species, such as tilapia (Araújo et al. 2016; Araújo et al. 2017). It is possible that some fish species, including tambaqui, respond to low P content by developing physiological mechanisms to utilize phytic phosphorus (Pfit) from plant‐based ingredients (Ellestad et al. 2002; Araújo et al. 2017; Sugiura 2018). Additionally, plant‐based diets may naturally contain phytase, contributing to the P utilization in some species. Ingredients such as wheat bran, corn, and rice are reported to present enzyme activity (Liu et al. 1998), improving mineral availability and reducing P discharge into the environment. However, it is most likely that this ingredient‐origin phytase is destroyed during feed processing. Therefore, it can be inferred that tambaqui in the final rearing stage does not require inorganic P supplementation when fed plant‐based diets, possibly due to its capacity to utilize phytate‐P. This may explain the performance data and the high phosphorus digestibility observed in this study with tambaqui. Nonetheless, P utilization/absorption is usually increased in P‐deficient animals due to a physiological adaptation of the gut and kidney cells to improve P uptake by increasing the expression of NAPi+ transporters, thus increasing P absorption and reducing P excretion (Chen et al. 2018). This mechanism may partially explain the efficiency observed in tambaqui fed lower dietary P. However, this hypothesis requires further investigation, as no studies have evaluated endogenous phytase activity or transporter expression in tambaqui. It is also important to note that the assumption of a fixed availability of phosphorus may introduce bias, as phosphorus digestibility can vary depending on dietary levels, physiological regulation, and endogenous losses. Thus, while tambaqui appears to utilize dietary P under the conditions tested efficiently, direct interspecies comparisons should be made with caution and under standardized methodologies.

Although the phosphorus utilization rate (PUR) is a commonly reported parameter in studies on mineral nutrition, it should be interpreted cautiously, particularly when phosphorus intake exceeds physiological requirements. As a ratio of weight gain to phosphorus intake, PUR naturally decreases as dietary phosphorus increases, even when growth remains stable. This mathematical decline does not necessarily indicate a reduction in biological efficiency but rather an overabundance of nutrient intake. Therefore, the PUR values in this study were used descriptively to illustrate the diminishing efficiency of phosphorus retention across treatments, rather than as a direct indicator of requirement. This approach aligns with previous studies that presented PUR alongside physiological outcomes to contextualize nutrient utilization (Antony Jesu Prabhu et al. 2013; Lu et al. 2017; Menezes et al. 2021).

Besides efficiency ratios, the body composition of fish can be a good indicator of nutrient intake and physiological well‐being. In this study, AP supplementation affected body fat deposition and moisture content in tambaqui. The increase in body fat deposition in fish fed low levels of dietary phosphorus has been extensively reported (Antony Jesu Prabhu et al. 2013; Sun et al. 2018; Zafar and Khan 2018; Menezes et al. 2021; Li et al. 2022). The increased adiposity in fish fed low P levels may be a result of decreased β‐oxidation, as insufficient inorganic phosphate leads to inhibition of the TCA cycle and accumulation of acetyl‐CoA, resulting in lower utilization of lipids as an energy source (Roy and Lall 2003). It may also be related to increased lipogenesis. Some studies have demonstrated that P deficiency causes alterations in enzymes of the pentose phosphate pathway in the hepatopancreas of fish (Onishi et al. 1981) and an increase in the activity involved in the synthesis of fatty acids, such as 3‐hydroxy‐3‐methylglutaryl‐coenzyme A reductase (HMGR) and up regulating the gene Elovl6 (Elongation of Very Long Chain Fatty Acids Protein 6) (Lu et al. 2017). These authors argue that the increased adiposity is sustained by protein catabolism. However, in our study, low dietary P levels did not affect the amount of body protein. Similar results were observed in carp (*Carassius auratus gibelio* var. CASIII) (Xie et al. 2017), taimen (*Hucho taimen*) (Wang et al. 2017), and senegalese sole (*Solea senegalensis*) (Salas‐Leiton et al. 2017). Additionally, lipid accumulation in P‐deficient fish could be related to the impaired function of perilipins. Perilipins are a group of six proteins (PLIN 1‐6) involved in lipid droplet biology (Londos et al. 2005; Granneman et al. 2017). In higher vertebrates, perilipins must be phosphorylated to induce lipid catabolism in adipose and muscle tissues (Londos et al. 2005). Therefore, as reported in mammals, P deficiency may impair lipid mobilization in fish. However, perilipin biology has been sparsely studied in fish, and although the genes and proteins have been identified in some fish species, no physiological roles have been demonstrated yet (Granneman et al. 2017).

Fish can use body phosphorus reserves to meet metabolic needs (Baeverfjord et al. 2019). The dietary AP levels did not affect phosphorus deposition in the tambaqui scales in this study. The fish could maintain phosphorus deposition in this tissue after 180 days of diet feeding without AP supplementation. The phosphorus content in the whole body is a relevant indicator of bone health in fish (Roy and Lall 2003; Lellis et al. 2004; Fontagné et al. 2009; Antony Jesu Prabhu et al. 2013; Deschamps et al. 2016; Fjelldal et al. 2016; Araújo et al. 2017; Menezes et al. 2021). The ash content in the vertebrae and scales is highly correlated (Deschamps et al. 2016; Menezes et al. 2021). Bones and scales share some regulatory pathways, with scales being highly responsive to dietary phosphorus deficiency (Roy and Lall 2003; Deschamps et al. 2016). However, tambaqui has various mineralized structures in its body, which may interact with mineral mobilization dynamics (Menezes et al. 2021). Thus, phosphorus stores in scales may only mobilize if these animals lack sufficient phosphorus for vital processes, since a limited effect of AP on the mineral content of scales was generally observed in this study. In contrast, a previous study of our group observed a profound effect of dietary P on different mineral body compartments of tambaqui (Menezes et al. 2021). Despite this, the mechanisms that allow for the differential regulation of bones and scales remain unknown but may be explained by developmental, chronological, and functional particularities (Deschamps et al. 2016), such as the age and sex of the fish and the cellular profile of mineralized structures. It is worth noting that fish in this trial started with ~ 400 g, which should be sufficient to develop all mineralized structures and could be responsible for the limited effect on the mineral content of different tissues and the growth performance. Therefore, comparative studies on the dynamics of mineral turnover among mineralized tissues and their relative contribution in response to phosphorus deficiency deserve further investigation.

In the present study, the tissues most affected by low dietary phosphorus levels, besides the whole body, were the vertebrae and opercular bones. These results were previously observed in tambaqui (Menezes et al. 2021), as well as in other fish species such as *Micropterus salmoides* (Wang et al. 2022), *Melanogrammus aeglefinus L*. (Roy and Lall 2003), and *Oncorhynchus mykiss* (Deschamps et al. 2016). These bony structures can respond to dietary phosphorus deficiency by releasing phosphorus for vital metabolic functions. The trend of reducing the Ca:P ratio in different mineral body compartments (except for the scales) highlights this hypothesis which seems that tambaqui might have an optimum Ca:P ratio for bone mineralization near to 1:1 according to results observed in this study and previous publication (Araújo et al. 2016; Menezes et al. 2021). In dietary phosphorus deficiency, calcium phosphate is mobilized from mineralized structures to prioritize metabolic and physiological processes (Baeverfjord et al. 2019). These results are supported by the high levels of plasma alkaline phosphatase in fish‐fed diets with low phosphorus levels found in this study. In higher vertebrates, this enzyme is used as an indicator of bone formation, as during bone formation and turnover, osteoblasts secrete high levels of alkaline phosphatase (Sugiura 2018).

A recent study with juvenile tambaqui demonstrated that fish‐fed diets deficient in AP showed decreased mineralization in various bone structures, primarily affecting the cranial and opercular bones (Menezes et al. 2021). This could be related to multiple mineralized structures in the tambaqui's body, such as scales, serrations, teeth, operculum, and fins. Therefore, phosphorus deficiency affects mineralized tissues differentially based on their physiological and biomechanical roles (Deschamps et al. 2016). This may explain the utilization of cranial and opercular bones to buffer the phosphorus imbalance in tissues and delay the negative impacts on growth. Additionally, larger fish, such as those in this study, have higher bone density and fully developed mineralized structures in their bodies, including scales, fins, vertebrae, ribs, cranial bones, and operculum (Baeverfjord et al. 2019) which might have affected the outcomes of this study and made comparisons with the data from other growth stages difficult.

Consequently, the utilization of phosphorus for bone turnover maintenance in these animals may be higher. However, there is no direct correlation between the degree of bone mineralization and bone quality. In salmonids subjected to prolonged dietary phosphorus deficiency, it was observed that even demineralized bone could continue to grow functionally and has the capacity for remineralization (Witten et al. 2018). Furthermore, providing absolute values to identify acceptable levels of mineralization in a particular species would require a standardization of methods for bone tissue analysis (Deschamps et al. 2016). Therefore, further studies are needed to determine how much tissue demineralization may be relevant for species like tambaqui.

Serum proteins serve as a primary index for evaluating fish's nutritional status, innate immune response, and overall health (Mazandarani and Hoseini 2017; Devi et al. 2019). Reduced levels of these proteins may be correlated with immunosuppression in fish (Avazeh et al. 2021). In the present study, total serum protein increased to 6.06 g/kg AP, while the highest albumin concentration was observed in fish fed with AP levels of 8 and 9.1 g/kg. This may indicate that fish fed with these AP levels were in a condition of physiological well‐being. Similar results were observed previously for tambaqui (*Colossoma macropomum*) (Araújo et al. 2017; Menezes et al. 2021) and carp (*Arisichthys nobilis*) (Li et al. 2022).

A deficiency of phosphorus (P) has been shown to compromise the immune functions of fish by reducing the function of physical barriers and the activity or content of antimicrobial substances (Chen et al. 2017, 2018; Gupta et al. 2023). The immune function of fish largely depends on antimicrobial substances such as immunoglobulins, lysozyme, and cytokines (Tort et al. 2003). These proteins mediate immune processes such as phagocytosis, opsonization, complement system activation, and pathogen neutralization (Tort et al. 2003). This study observed the highest lysozyme activity in tambaqui‐fed diets containing 5.8 and 8 g/kg AP. A similar result was observed for the total immunoglobulin level in tambaqui in this study, which increased to 6.17 g/kg AP. The increase in total immunoglobulins and lysozyme levels is strongly associated with enhanced humoral and innate immunity, which may lead to disease prevention in fish (Gupta et al. 2023). Therefore, the results observed in this study suggest that a higher AP level in the diet compared to the requirement for maximum growth may promote immune response and help prevent infections and diseases in tambaqui compared to the level needed to maintain average growth.

The serum Pi (inorganic phosphorus) levels of tambaqui in this study were unaffected by AP supplementation. Serum Pi levels are known to reflect recent dietary intake of the mineral rather than the P status of the fish (Antony Jesu Prabhu et al. 2013; Araújo et al. 2017; Menezes et al. 2021). This study established a fasting period of 24 h before blood collection to minimize this effect. Therefore, the use of serum Pi values has been questioned by some researchers as a reliable response variable to indicate the adequacy of dietary P levels for fish (Roy and Lall 2003; Yao et al. 2014; Araújo et al. 2017). Altogether, this affected the outcomes observed for this parameter in this study.

The findings of this study indicate that tambaqui in the grow‐out stage could maintain growth without dietary inorganic P supplementation to plant‐based diets. A minimum of 4.1 g/kg (or 25.2 mg AP/kg BW^0.8^/day) is needed to maintain growth parameters. However, this leads to higher adiposity in the fish. On the other hand, the phosphorus requirement for maximizing bone mineralization was 10.3 g/kg AP (or 63.5 mg AP/kg BW^0.8^/day). In comparison, the use of 6.17 g/kg AP (or 37.9 mg AP/kg BW^0.8^/day) appears sufficient to promote better immune response parameters such as total serum proteins, lysozyme, and immunoglobulins, thereby influencing the fish's resistance.

## Author Contributions

**Ludmila L.C. Menezes:** formal analysis, investigation, methodology, project administration, writing – original draft preparation, visualization, data analysis; **Cristielle N. Souto:** investigation, formal analysis, visualization, methodology, data analysis; **Vânia M.V. Machado:** investigation, visualization, supervision, writing – review and editing; **Danilo C. Proença:** visualization, data analysis, writing – review and editing; **Guilherme W. Bueno:** conceptualization, funding acquisition, visualization, validation, writing – review and editing; **Igo G. Guimarães:** conceptualization, funding acquisition, supervision, visualization, validation, data analysis, writing – review and editing.

## Ethics Statement

The authors confirm that the ethical policies of the journal, as noted on the journal's author guidelines page, have been adhered to and the appropriate ethical review committee approval has been received (Animal Ethics Committee of the Universidade Federal de Jataí – process number 014/2020 CEUA‐UFJ).

## Conflicts of Interest

The authors declare no conflicts of interest.

## Supporting information

Appedix 1 Menezes et al Revised.

## Data Availability

The data that support the findings of this study are available from the corresponding author upon reasonable request. The corresponding author can provide the data upon reasonable request.
