# Supplementary material for: Dietary Phosphorus Requirement for Tambaqui, Colossoma macropomum, in the Grow‐Out Phase
Source: J Anim Physiol Anim Nutr (Berl). 2025 Jul 17;109(6):1321–32. doi: 10.1111/jpn.70005 (PMC12644302; doi:10.1111/jpn.70005)
Supplement: Supplementary file 1 — Appedix 1 Menezes et al Revised. [file JPN-109-1321-s001.docx]

**Digestibility trial for determining the digestible P content of experimental diets**

A total of 90 fish (532±25g) were randomly assigned into 15 500 L-aquaria to determine the apparent digestibility coefficients (ADC) nutrients of the experimental diets. Experimental diets for the digestibility study were produced similarly as described in section 2.1 of Materials and Methods except for the inclusion of chromium oxide III at 2 g/kg in all diets at the expense of the kaolin. These aquaria were connected to a water recirculation system equipped with a biological and mechanical filter and used for the feeding procedure. Diets were then assigned to the aquaria, and the fish underwent a 7-day adaptation period, during which they were fed three times daily to apparent satiety.

After the adaptation period, five groups of fish (from each dietary treatment) were transferred to feces collection tanks 30 minutes after their last feeding. Feces were collected by sedimentation using five 300 L conical-bottomed aquaria as previously reported (Guimarães et al. 2008; da Mota et al., 2015). After transferring, feces were collected in 30 minutes interval for 6 hours as previously recommended by da Mota et al. (2015) for reducing P leaching from feces to the water. Then, the groups of fish returned to their respective feeding tanks. External aeration was provided to the collection tanks via an air blower diffused with air stones. After each feces collection, the tanks were drained, thoroughly washed with running water, and refilled with fresh water to prevent potential contamination. On the other day, another 5 groups of fish from each dietary treatment were transferred and the same procedure was repeated. This process was repeated for the 15 groups of fish until the required fecal samples were obtained for analysis.

Water quality parameters, including dissolved oxygen, ammonia, nitrite, and pH, were monitored weekly using Alcon Labcon colorimetric tests, following the manufacturer's instructions. A mercury bulb thermometer was used to measure system temperature. During the experimental period, dissolved oxygen levels remained above 8 mg/L, total ammonia was 0.25 mg/L, nitrite levels ranged from 0.0 to 0.25 mg/L, pH values ranged from 6.8 to 7.2, and the temperature was maintained between 27.1°C and 29.8°C. All parameters were kept within the optimal range for the species.

After collecting, feces were dehydrated in an air-forced oven (55°C for 48 hours), ground, and stored at -20°C. The ADC of nutrients of the diets was calculated using the following equation (Cho & Slinger, 1985): ADC(d)=100-[100 (%Cr_2_O_3_feed/%Cr_2_O_3_feces) x (%Nfeces/%Nfeed)], in which ADC = apparent digestibility coefficient; Cr_2_O_3_feed = % chromium-III oxide in feed; Cr_2_O_3_faeces = % chromium-III oxide in feces; Nfeed =nutrients in feed; Nfeces = nutrients in feces.

Dry matter, crude protein and phosphorus content of the samples of feces and diets were determined according to the description in section 2.3 of Materials and Methods, while chromium oxide was determined according to Bremer Neto et al. (2005).

###

### **Appendix 1**. Apparent digestibility coefficient (ADC) of dry matter, crude protein, and phosphorus in tambaquis fed different levels of available phosphorus in the diet (n = 3)

|  | **Levels of available phosphorus (g/kg)** | | | | | |  |
| --- | --- | --- | --- | --- | --- | --- | --- |
| **Variables** | **4.1** | **5.8** | **8.0** | **9.1** | **10.3** | **P value** | |
| **ADC dry matter (%)** | 66.63  ±2.2 | 67.21  ±0.9 | 64.72  ±0.5 | 61.86  ±0.7 | 65.00  ±0.4 | 0.34 | |
| **ADC crude protein (%)** | 94.35  ±0.9 | 89.70  ±0.6 | 82.59  ±0.4 | 91.09  ±1.04 | 89.90  ±1.3 | 0.93 | |
| **ADC total phosphorus (%)^1^** | 83.48  ±1.4 | 81.89  ±0.6 | 83.35  ±0.8 | 79.07  ±2.6 | 76.79  ±1.3 | 0.014 | |

^1^Linear= 53.25688-0.0073558x (R^2^= 0.94)
